# Insulinoma: A Recurrent Pancreatic Tumor Amendable to Computed Tomography–Guided Ethanol-Lipiodol Injection

**DOI:** 10.14309/crj.0000000000000539

**Published:** 2021-02-26

**Authors:** Himesh B. Zaver, Khaled I. Alnahhal, Hassan Ghoz, Ricardo Paz-Fumagalli, Massimo Raimondo, Frank J. Lukens

**Affiliations:** 1 Department of Internal Medicine, Mayo Clinic, Jacksonville, FL; 2 Division of Vascular Surgery, Mayo Clinic, Jacksonville, FL; 3 Division of Gastroenterology and Hepatology, Mayo Clinic, Jacksonville, FL; 4 Department of Radiology, Mayo Clinic, Jacksonville, FL

## Abstract

Insulinomas are rare, with an annual incidence of 1–4 people per mission. Insulinomas are the most common functioning endocrine neoplams of the pancreas. Endoscopic ultrasound has both diagnostic and therapeutic yield in undifferentiated pancreatic tumors. We present a case of a recurrent insulinoma, refractory to surgical and medical management diagnosed with endoscopic ultrasound. Our case uniquely conveys a successful, alternative approach to addressing symptomatic insulinomas refractory to surgical or medical management through computed tomography–guided percutaneous ethanol-lipiodol injection.

## INTRODUCTION

Insulinomas are a rare neuroendocrine tumor of the pancreas. We present a unique case of a recurrent insulinoma refractory to surgical and medical management successfully treated with computed tomography (CT)-guided percutaneous ethanol-lipiodol injection.

## CASE REPORT

**Figure 1.**
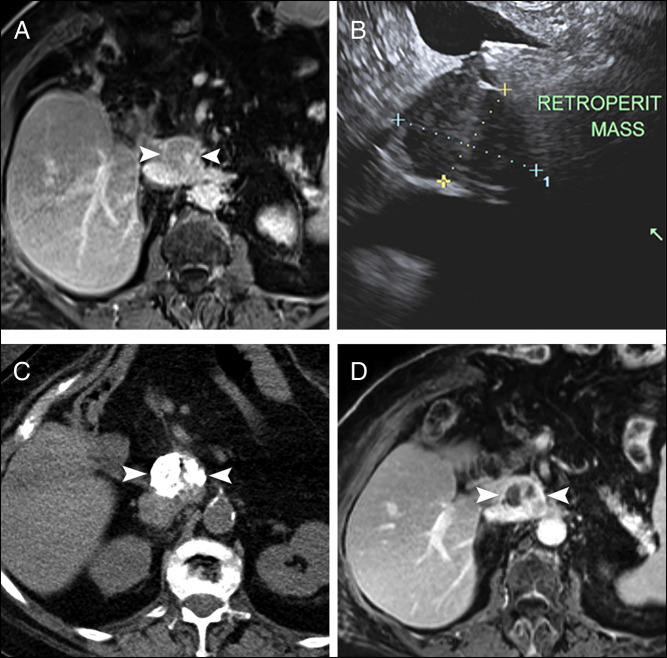
(A) Endoscopic ultrasound significant for irregular, retroperitoneal mass. Fine needle aspiration with biopsy positive for malignancy consistent with a recurrent, metastatic, well-differentiated neuroendocrine tumor. Immunohistochemical staining was positive for chromogranin and synaptophysin. (B) Contrast-enhanced magnetic resonance imaging shows an enlarged, uniformly enhancing portocaval lymph node metastasis (arrowheads) from pancreatic neuroendocrine tumor. (C) Computed tomography image after percutaneous injection of lipiodol/ethanol mixture shows extensive distribution of the high radiodensity material within the metastatic lymph node (arrowheads). (D) Contrast-enhanced magnetic resonance imaging 3 months after treatment shows extensive zones of lack of enhancement within the treated metastatic lymph node (arrowheads), indicating tumor necrosis.

An 81-year-old woman presented to our tertiary care center with persistent episodes of hypoglycemia during which she reports palpitations, diaphoresis, and confusion. The patient's medical history was significant for recurrent hypoglycemia initially because of a 2.5-cm insulinoma at the pancreatic head diagnosed 15 years earlier at an outside hospital amendable to enucleation therapy. Symptom recurrence 7 years after the initial diagnosis was significant for a 2-cm highly differentiated neuroendocrine tumor located at the previous surgical bed treated with a pyloric-preserving pancreatoduodenectomy (Whipple procedure). She was symptom-free for another 7 years before symptom reoccurrence for the third time. Repeat endoscopic ultrasound (EUS) with fine-needle aspiration (FNA) was nondiagnostic (no pancreatic body/tail mass or cyst), and FNA of a peripancreatic lymph node was negative for neoplastic immunohistochemistry (negative keratin, chromogranin, and synaptophysin), prompting referral to our pancreas clinic. With concern of reoccurrence of insulinoma, workup was significant for C-peptide 4.5 ng/mL, proinsulin 302 pmol/L, and abdominal magnetic resonance imaging (MRI) found a 3.8-cm heterogeneous mass posterior and caudal to the pancreatic anastomosis from previous Whipple procedure (Figure 1A). The patient was placed on diazoxide (pancreatic β-cell inhibitor), and it was decided to proceed with repeat EUS with FNA (Figure 1B). FNA biopsy was significant for malignancy consistent with a recurrent, metastatic, well-differentiated neuroendocrine tumor. Immunohistochemical staining was positive for chromogranin and synaptophysin.

The retroperitoneal location and size of the recurrent insulinoma posed a clinically difficult situation to address. After multidisciplinary goals of care conversation involving the patient, it was decided to focus on symptom management instead of tumor removal. Medical management was not considered because of symptom reoccurrence. EUS with radiofrequency ablation (RFA) was not considered because of the size of the mass and preference of chemical ablation. Surgical debulking was not an option because of the tumor's retroperitoneal location and patient's altered postsurgical anatomy. Interventional radiology conveyed that CT-guided percutaneous ethanol-lipiodol injection of the insulinoma was an option that has been used to treat other types of tumors in the past. It was decided to proceed with CT-guided percutaneous ethanol-lipiodol injection (Figure 1C; 10 cc of 1:4 lipiodol:ethanol). The procedure was successful, and the patient was discharged hemodynamically stable without complications. At the 3-month follow-up, the patient reported complete resolution of previous symptoms and was off diazoxide. Repeat abdominal MRI at this time showed radiographic evidence of tumor necrosis (Figure 1D). The patient continues to have close follow-up with oncology and endocrinology.

## DISCUSSION

The majority of insulinomas follow the “90% Rule” in which the majority are benign (90%), solitary (90%), < 2 cm (90%) in diameter, and equally distributed within the head, body, and tail of the pancreas.^1–3^ Our patient's tumor was recurrent and >2 cm on multiple admissions despite enucleation, Whipple procedure, and medical management with diazoxide.

Detection methods for undifferentiated pancreatic masses are broad. Initial laboratory workup including serum insulin, c-peptide, and glucose challenge testing often leads to noninvasive imaging such as CT or MRI. However, endoscopy, in particular EUS, plays a significant role in diagnosis.^1–3^ EUS-FNA has better sensitivity (86.8%) and specificity (95.8%) compared with conventional MRI or CT with regards to diagnosing pancreatic tumors.^4^ After localization of an insulinoma, surgical management is considered first-line therapy. Candidacy for surgery is mediated on size, location, and type. Enucleation of smaller masses or partial pancreatectomy of masses located within the body or tail of the pancreas is ideal to preserve pancreatic parenchyma and exocrine/endocrine function.^1,2^ Our patient, before referral to our center, had trialed enucleation and pyloric-preserving pancreatoduodenectomy with subsequent recurrence. Recurrence of insulinomas is reported to be between 5% and 20% with a higher rate of recurrence in patients with multiple endocrine neoplasia 1.^1,3,5^ Our patient had recurrence at 7 and 15 years after initial diagnosis, and she did not have clinical features concerning for a concurrent pituitary adenoma or hyperparathyroidism suspicious of multiple endocrine neoplasia 1.

In patients who are not surgical candidates or in patients who require a bridge before invasive treatment, medical management in the form of diazoxide, a beta-cell receptor antagonist or somatostatin analogs such as octreotide and lanreotide can be used.^1,6^ Our patient was initially placed on diazoxide with symptoms refractory despite medical and surgical management. Because of the retroperitoneal location of the insulinoma and altered surgical anatomy from her previous Whipple procedure, it was deemed that she would not make an ideal surgical candidate because of perioperative risks.

Successful techniques other than surgery have been described in previous literature, including EUS-guided and CT-guided alcohol ablation, RFA, and embolization of an insulinoma.^7–12^ Alternative approaches, especially EUS-guided approaches, should be kept in mind by gastroenterologists and advanced endoscopists alike. EUS-guided approaches are relatively new compared with CT-guided ablation, which has been practiced for over 30 years for insulinomas, hepatic neoplasms, and medullary thyroid carcinoma.^10,13,14^ EUS-guided ethanol-lipiodol injection would have been an equally viable option given the successful procurement of tumor tissue through EUS; however, CT-guided ablation was used in our case based on patient preference and clinical experience of the interventional radiology team with regards to CT-guided chemical ablation.

In the setting of a recurrent, retroperitoneal pancreatic insulinoma complete elimination of the tumor burden was ancillary to symptom management, and control of her symptomatic hypoglycemia was the main objective. Our patient had successful treatment through CT-guided ethanol-lipiodol injection of her insulinoma with complete resolution of her symptomatic episodes. In conclusion, EUS- and CT-guided alcohol or RFA embolization offers a less invasive alternative to surgical intervention and should be considered in patients with refractory or poorly localized pancreatic tumors. Our case uniquely conveys a successful, alternative approach to addressing symptomatic insulinomas refractory to surgical or medical management through CT-guided percutaneous ethanol-lipiodol injection.

## DISCLOSURES

Author contributions: All authors contributed equally to this manuscript. HB Zaver is the article guarantor.

Financial disclosure: None to report.

Informed consent was obtained for this case report.
